# Sleep Disorders and Associated Factors Among Medical Students in Saudi Arabia: A Systematic Review and Meta-Analysis

**DOI:** 10.7759/cureus.100414

**Published:** 2025-12-30

**Authors:** Hassan Zaher M. Alqarni, Elham M Alqarni, Mezyed Fahad Alghanim, Mohd Ali Aldowaher, Waleed Mohammed Alshehri, Maram Ali M. Alshahrani, Malik Homoud S. Alshahrani, Lama Ali I. Asiri, Mohammed Shari Alshahrani

**Affiliations:** 1 Family Medicine, Aseer Central Hospital, Abha, SAU; 2 Faculty of Medicine, King Khalid University, Abha, SAU; 3 General Practice, College of Medicine, King Saud bin Abdulaziz for Health Sciences, Riyadh, SAU; 4 General Practice, College of Medicine, Almaarefa University, Riyadh, SAU; 5 Medical Physics, King Abdulaziz University Faculty of Medicine, Jeddah, SAU; 6 General Practice, College of Medicine, King Khalid University, Abha, SAU; 7 Diagnostic Radiology, Armed Forces Hospital of Southern Region, Khamis Mushait, SAU

**Keywords:** excessive daytime sleepiness, insomnia, kingdom of saudi arabia (ksa), medical students, sleep disorders

## Abstract

Sleep disorders are increasingly recognized among medical students in Saudi Arabia, a population facing demanding academic schedules, psychological stress, and lifestyle factors that negatively influence sleep health. This systematic review and meta-analysis aimed to synthesize existing evidence on the prevalence of sleep disorders in this group and to identify associated demographic, behavioral, and psychological factors. A comprehensive search of PubMed, Scopus, Web of Science, and Google Scholar identified observational studies assessing insomnia, excessive daytime sleepiness, and general sleep disturbances among medical students in Saudi Arabia. Eleven studies comprising a total of 3,874 participants met the inclusion criteria. Validated sleep assessment tools were used across all studies, although reported prevalence varied widely. Insomnia rates ranged from 32 percent to 86.8 percent, and excessive daytime sleepiness from 37.3 percent to 56.9 percent. Meta-analysis of five studies produced a pooled insomnia prevalence of 52 percent with substantial heterogeneity. Meta-analysis of three studies yielded a pooled prevalence of 44 percent for excessive daytime sleepiness, also with high heterogeneity. Factors consistently associated with sleep problems included female gender, younger age, preclinical academic level, smoking, high academic workload, and psychological distress such as anxiety and depression. Findings regarding academic performance were inconsistent, with some studies reporting poorer outcomes among students with sleep disorders while others found no relationship. Overall, the evidence indicates that sleep disorders are highly prevalent among medical students in Saudi Arabia and are influenced by multiple interacting factors. Interventions focused on sleep hygiene, stress reduction, and mental health support may improve student well-being and academic functioning.

## Introduction and background

Sleep disorders encompass a broad spectrum of conditions, including insomnia, excessive daytime sleepiness, circadian rhythm disruptions, and sleep-related breathing abnormalities [1]. These problems have gained increasing attention as their global burden continues to rise, yet many affected individuals remain undiagnosed or undertreated [2]. Disturbed sleep is linked to adverse physical, cognitive, and emotional outcomes, reduced functional performance, and a heightened risk of psychiatric and chronic medical conditions, which together create a substantial societal impact [3]. Recent international evidence has emphasized the scale of this issue, with a comprehensive systematic review indicating that obstructive sleep apnea affects nearly 46 percent of adults worldwide, followed by poor sleep quality at about 40 percent, other sleep disturbances at 37 percent, insomnia at approximately 29 percent, and excessive daytime sleepiness at 19 percent [4].

Medical students are widely recognized as a population vulnerable to significant sleep disruption due to demanding coursework, prolonged study hours, examinations, clinical responsibilities, and exposure to stress [5]. Studies consistently show that medical students experience higher levels of sleep disturbance compared with non-medical peers and the general population [6,7]. Poor sleep in this group can influence learning capacity, clinical judgment, attention, and emotional stability, and may increase vulnerability to anxiety and depression [8]. However, sleep health among medical students is often shaped by contextual factors, including educational structure, social habits, and cultural expectations, leading to considerable variation across countries [9].

In Saudi Arabia, research on sleep disorders among medical students has increased, yet findings remain fragmented across individual studies that vary in design and measurement tools. Prevalence estimates differ widely, with some studies reporting insomnia in more than two-thirds of students and others documenting excessive daytime sleepiness in over one-third [10,11]. Several consistent risk factors have emerged, including female gender, younger age, preclinical academic standing, smoking, heavy academic workload, and psychological distress [12,13]. Associations with academic performance vary, with some studies reporting poorer outcomes among students with sleep disturbances and others finding no meaningful relationship.

Given these inconsistencies, a synthesized overview is needed to better understand the magnitude of sleep disorders among medical students in Saudi Arabia and the factors associated with them. This systematic review and meta-analysis aims to provide pooled estimates of insomnia and excessive daytime sleepiness and to summarize key demographic, behavioral, and psychological risk factors identified in the literature.

## Review

Methods

Study Design

This systematic review and meta-analysis were conducted to synthesize evidence on the prevalence of sleep disorders, their associated factors, and potential academic implications among medical students in Saudi Arabia. The review followed the methodological guidance of the Preferred Reporting Items for Systematic Reviews and Meta-Analyses (PRISMA) 2020 statement and the Cochrane Handbook for Systematic Reviews of Interventions [14,15]. The research question was formulated using the PICO (Population, Intervention, Comparison, Outcome) framework [16], defining the population as undergraduate medical students enrolled in institutions across Saudi Arabia and the exposure as the presence of sleep disorders or sleep-related disturbances. Comparisons involved students without such disturbances or those differing in severity levels. The outcomes of interest included the prevalence of sleep disorders as well as demographic, behavioral, psychological, and academic factors associated with these conditions.

Search Strategy

A comprehensive search of published literature was conducted across PubMed (MEDLINE), Scopus, Web of Science, and Google Scholar from database inception through December 2025. The search strategy combined controlled vocabulary terms (e.g., MeSH where applicable) with free-text keywords related to sleep disorders, medical students, and Saudi Arabia. Boolean operators (AND/OR) were used to ensure broad sensitivity and capture all potentially relevant studies. Filters were applied to include only English-language publications involving human participants. Because Google Scholar does not allow reproducible advanced filtering, the first four pages of search results of approximately 40 records were screened, as the platform typically ranks the most relevant studies at the top. Additionally, reference lists of eligible articles were checked manually to identify any relevant studies missed by database searches. Detailed search strings for each database are provided in the Supplementary Appendix.

Eligibility Criteria

Studies were included if they focused on medical students enrolled at universities within Saudi Arabia and reported quantifiable outcomes related to sleep disorders, sleep quality, insomnia, excessive daytime sleepiness, or related sleep disturbances. Only observational study designs, such as cross-sectional, cohort, or case-control studies, were eligible. Articles were limited to English-language publications involving human participants. Studies were excluded if they were conducted outside Saudi Arabia, included populations other than medical students without separable subgroup data, or were non-original works such as reviews, commentaries, conference abstracts, editorials, or case reports. Research that did not clearly define or measure a sleep-related outcome was also excluded. When multiple articles appeared to use overlapping datasets, the version with the most complete methodology or the most recent results was retained.

Study Selection

**Figure 1:**
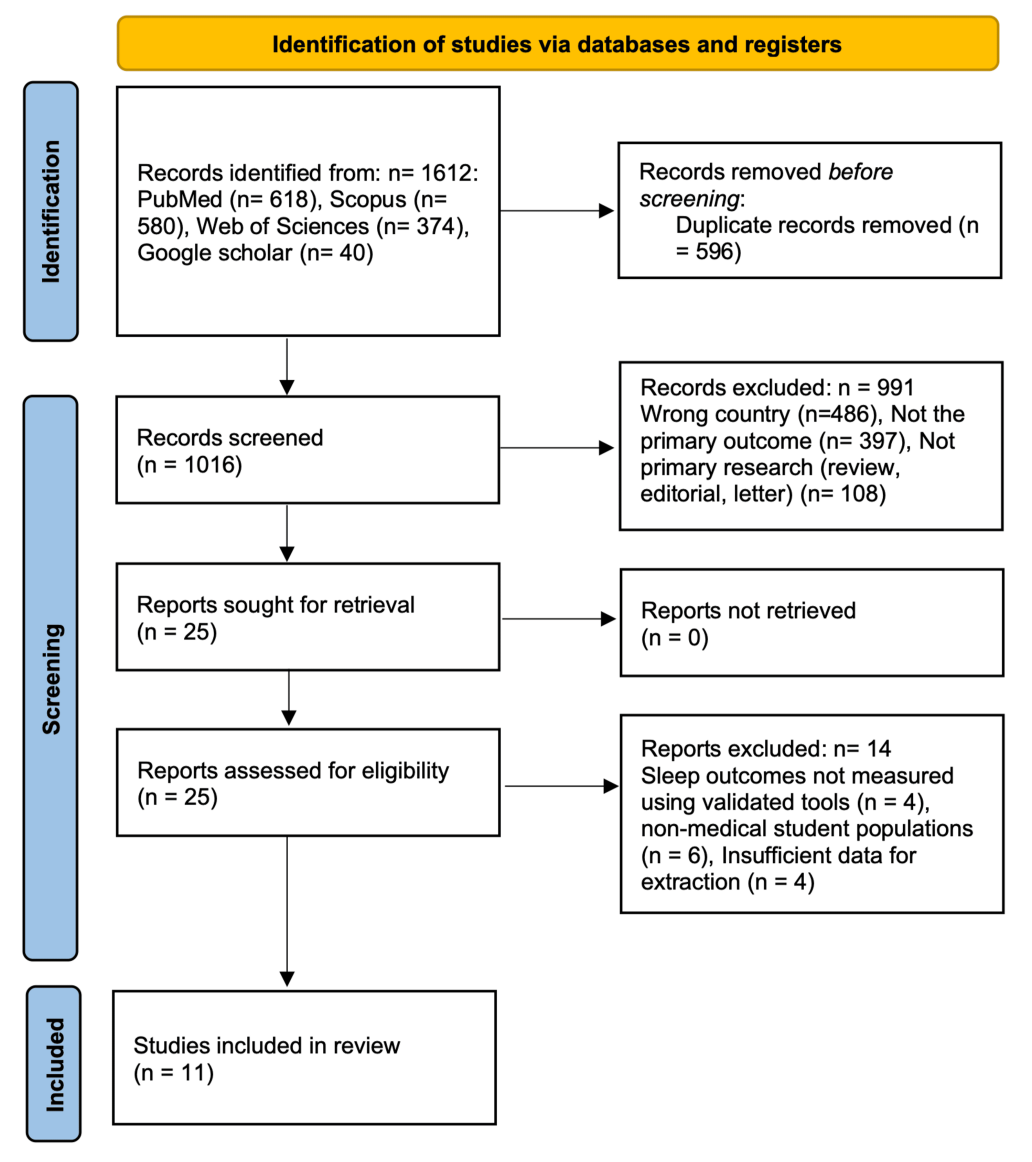
PRISMA flow diagram illustrating the selection process for studies included in the systematic review This flow diagram follows the PRISMA 2020 guidelines as described by Page et al. [15]. PRISMA: Preferred Reporting Items for Systematic Reviews and Meta-Analyses

All identified citations were imported into Rayyan for organization and duplicate removal (Rayyan, Cambridge, USA) [17]. Screening was conducted in two stages: title and abstract review, followed by a full-text assessment of potentially relevant articles. Four reviewers evaluated the articles independently according to the predefined eligibility criteria. Any discrepancies were resolved through team discussion or consultation with a senior reviewer. The study identification and selection process is illustrated in the PRISMA flow diagram (Figure 1).

Data Extraction

Data extraction was performed independently by four reviewers using a standardized spreadsheet to ensure consistency in collected information. Extracted variables included study characteristics (authors, publication year, geographic location, study design, sample size), participant demographics, the sleep assessment tools used, prevalence estimates for the sleep disorder of interest, reported risk factors, and measures related to academic performance, such as grade point average. When needed, authors of the original studies were contacted for clarification. All extracted data were compared across reviewers, and discrepancies were resolved by consensus.

Quality Assessment

The methodological quality of the included studies was appraised using the Newcastle-Ottawa Scale (NOS) adapted for cross-sectional research designs [18]. This tool assesses the adequacy of participant selection, control for potential confounding factors, and the robustness of outcome or exposure measurement. Each study could receive a maximum of nine points, with scores classified as high, moderate, or low quality. The assessment was conducted independently by four reviewers, and any differences in scoring were resolved through discussion to achieve consensus.

Statistical Analysis

All statistical procedures were conducted using R software (version 4.3.3; R Foundation for Statistical Computing, Vienna, Austria) with the meta and metafor packages. Random effects models were used to generate pooled prevalence estimates because variations in study design and measurement tools were expected across the included studies. Proportion data were synthesized using the Freeman-Tukey double arcsine transformation to stabilize variance [19]. Results were then back-transformed to the original proportion scale to facilitate interpretation. Heterogeneity among studies was evaluated using Cochran’s Q test and the I² statistic. Higher I² values were interpreted as evidence of substantial variability that was not attributable to sampling error. Subgroup analyses or meta-regression were not performed because the number of available studies and the inconsistency in measurement approaches limited meaningful stratification. Given that this review synthesized prevalence estimates derived from different screening instruments, populations, and academic settings, substantial heterogeneity was anticipated. Therefore, random-effects models were selected a priori as the most appropriate approach for pooling prevalence estimates. Although high I² values were observed, these were interpreted in the context of known methodological and clinical diversity rather than as evidence of invalid pooling.

Assessment of publication bias was not performed because the individual meta-analyses included fewer than 10 studies (five studies for insomnia and three studies for excessive daytime sleepiness), which is below the recommended threshold for a reliable evaluation of funnel plot asymmetry [20]. Statistical tests such as Egger’s regression and Begg’s rank correlation have limited power with small numbers of studies and may yield misleading or unstable results [21]. Therefore, publication bias assessment was intentionally omitted for both pooled analyses. Statistical significance for all analyses was set at p < 0.05.

Results

Study Selection

Figure 1 presents the flow of study identification, screening, and inclusion based on the database search and eligibility assessment. The search yielded 1,612 records across PubMed, Scopus, Web of Science, and Google Scholar. After removing 596 duplicates, 1,016 unique records were screened. A total of 991 records were excluded during title and abstract screening because they were conducted in countries outside Saudi Arabia, did not address sleep-related outcomes, or were not primary research articles. Twenty-five full-text reports were evaluated for eligibility, and all were successfully retrieved. Fourteen of these were excluded for reasons including the use of non-validated sleep measures, failure to focus on medical student populations, or insufficient extractable data. Ultimately, 11 studies met the inclusion criteria and were incorporated into the qualitative synthesis and meta-analysis.

Characteristics of Included Studies

A total of 11 studies met the inclusion criteria, all of which used cross-sectional designs and were conducted among medical or health sciences students in Saudi Arabia (Table 1). Sample sizes ranged from 129 to 576 participants, and all studies relied on validated self-reported questionnaires to assess sleep outcomes. Various tools were used, including the Athens Insomnia Scale, Epworth Sleepiness Scale, Pittsburgh Sleep Quality Index (PSQI), Insomnia Severity Index, Sleep-50 Questionnaire, and others. Although most studies were carried out in large urban centers such as Riyadh, Jeddah, Makkah, Majmaah, and Al-Jouf, one multicenter study did not specify individual cities within the Kingdom. Across studies, the primary outcomes focused on insomnia, sleep deprivation, excessive daytime sleepiness, general sleep disturbances, or multiple sleep disorder subtypes. Several investigations also explored academic performance and psychological factors such as anxiety, depression, and stress.

**Table 1:** Characteristics of included studies AIS = Athens Insomnia Scale; ESS = Epworth Sleepiness Scale; ISI = Insomnia Severity Index; PSQI = Pittsburgh Sleep Quality Index; SHAI = Short Health Anxiety Inventory; HADS = Hospital Anxiety and Depression Scale; K10 = Kessler Psychological Distress Scale; PHQ-9 = Patient Health Questionnaire-9; GPA = Grade Point Average; MSS = Medical Student Syndrome. All included studies used cross-sectional designs and relied on self-reported questionnaires. Only Saudi Arabian study locations are listed; if city information was unavailable, it was omitted.

Study (author, year)	Location (city)	Study design	Sample size	Population	Tools used	Main outcomes
Alshaaer et al., 2012 [11]	Riyadh	Cross-sectional	129	Medical students	Athens Insomnia Scale (AIS)	Insomnia severity, GPA
Al-Zahrani et al., 2016 [10]	Al-Kharj	Cross-sectional	161	Male medical students	Epworth Sleepiness Scale (ESS)	Excessive daytime sleepiness (EDS), GPA
AlQahtani et al., 2017 [22]	Riyadh	Cross-sectional	237	Medical students	ESS, sleep habits/ medication-use questionnaire	Sleep disorders, medication use, GPA categories
Ibrahim et al., 2017 [23]	Jeddah	Cross-sectional	576	Medical students	PSQI, ESS, HADS	Sleep quality, EDS, anxiety/depression
Almansour et al., 2020 [13]	Riyadh	Cross-sectional	430	Medical students	Sleep/sleep deprivation questionnaire	Sleep deprivation, causes, GPA
Mohamed et al., 2020 [24]	Majmaah	Cross-sectional	190	Medical students	ISI, State Anxiety Scale	Insomnia, anxiety
Goweda et al., 2020 [25]	Makkah	Cross-sectional	438	Medical students	Sleep-50 Questionnaire	Multiple sleep disorders
Rehab Ali Mohamed et al., 2020 [26]	Al-Jouf	Cross-sectional	303	Medical students	PSQI	Sleep quality, GPA
Alrashed et al., 2021 [12]	Riyadh	Cross-sectional	463	Clinical-year students & interns	ISI, K10, PHQ-9	Insomnia, psychological factors
Abdulghani et al., 2023 [27]	Riyadh	Cross-sectional	544	Health professions students	SHAI	MSS, anxiety, GPA
Alasimi et al., 2025 [28]	Various cities in KSA	Cross-sectional	403 (322 KSA students included)	Respiratory therapy students	ISI	Insomnia, GPA

Prevalence of Sleep Disorders and Excessive Daytime Sleepiness

The reported prevalence of sleep problems varied substantially across studies. Insomnia rates ranged from 32 percent to nearly 87 percent depending on the assessment tool and student population (Table 2). Poor sleep quality measured by the PSQI was common, with some studies reporting that more than two-thirds of participants met the cutoff for inadequate sleep. Excessive daytime sleepiness was also widely reported, ranging from 37.3 percent to 56.9 percent among studies using the Epworth Sleepiness Scale. Sleep deprivation was identified in approximately three-quarters of medical students in one study, reflecting the high burden of insufficient sleep within this population.

**Table 2:** Key findings and associated factors Prevalence values represent the proportion of students meeting the diagnostic cutoff for insomnia, sleep disorders, or excessive daytime sleepiness based on validated measurement tools used in each study. Reported risk factors and odds ratios (ORs) reflect statistically significant associations as described by the original study authors. “No GPA association” indicates no statistically significant relationship between sleep outcomes and academic performance. AIS = Athens Insomnia Scale; GPA = Grade Point Average; EDS = Excessive Daytime Sleepiness; MSS = Medical Student Syndrome; KSA = Kingdom of Saudi Arabia.

Study (author, year)	Prevalence of sleep disorder	Significant associated factors (with ORs when available)	Academic performance findings
Alshaaer et al., 2012 [11]	Insomnia prevalence not explicitly reported	– Insomnia severity measurable via AIS	Weak negative correlation with GPA (r = –0.163, p = 0.032)
Al-Zahrani et al., 2016 [10]	EDS: 37.8%	– No significant demographic associations	No significant GPA difference between EDS vs non-EDS
AlQahtani et al., 2017 [22]	Sleep disorders: 63.4% EDS: 56.9%	– 53.6% used anti-insomnia medications – No ORs reported	Medication users more likely to have excellent GPA (51.9%)
Ibrahim et al., 2017 [23]	Poor sleep: 70.4% EDS: 37.3%	– Female gender (p=0.02) – Younger age ≤21 yrs (OR = 2.4) – Preclinical years (aOR = 1.82) – Morbid anxiety strongest predictor (aOR = 3.92)	Poor sleep associated with higher GPAs (unique finding)
Almansour et al., 2020 [13]	Sleep deprivation: 74%	– Sleep 4–6 hrs common – Causes: assignments, stress, internet – No ORs reported	Significant association between sleep deprivation and lower GPA (p=0.003)
Mohamed et al., 2020 [24]	Insomnia: 70%	– Strong association with anxiety (p < 0.001) – Severe anxiety → insomnia in 91.5%	GPA not measured
Goweda et al., 2020 [25]	≥1 sleep disorder: 73.7% Narcolepsy: 51.6%	– Higher prevalence in females (p=0.001) – Higher screen time – No ORs reported	No significant association with GPA (p=0.484)
Rehab Ali Mohamed et al., 2020 [26]	Poor sleep: 86.8%	– Higher in 2nd year (p=0.009) – Living with family protective (p=0.05)	Poor sleepers had significantly worse academic performance (p<0.001)
Alrashed et al., 2021 [12]	Insomnia: 34.9%	– Female gender (OR = 1.67) – Interns (OR = 3.0) – Severe stress (OR = 2.81) – Severe depression (OR = 3.10) – Irregular rotations (OR = 3.37)	No direct GPA analysis
Abdulghani et al., 2023 [27]	MSS: 8.5%	– Females more anxious (p < 0.0001) – GPA decline linked to hypochondria (OR = 3.37) – Frequent MSS symptoms → hypochondria (OR = 2.93)	GPA decline (<0.20) significantly related to MSS
Alasimi et al., 2025 [28]	Insomnia: 32% (KSA)	KSA: – Female gender (p=0.003) – Smoking (p<0.001) – Lower GPA associated with higher ISI (p<0.001)	Low GPA associated with worse insomnia (KSA only)

Meta-Analysis of Insomnia Prevalence

**Figure 2:**
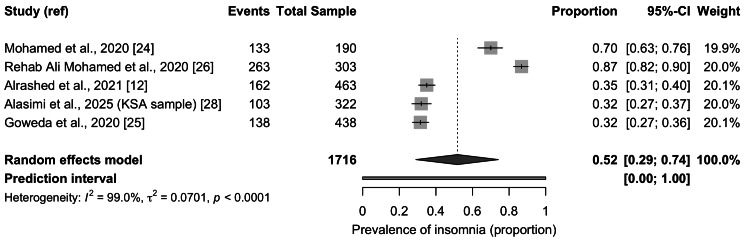
Pooled prevalence of insomnia among medical students in Saudi Arabia KSA = Kingdom of Saudi Arabia

Five studies were eligible for the pooling of insomnia prevalence. The combined sample included 1,716 students. The random effects model produced a pooled prevalence estimate of 0.52 with a 95 percent confidence interval from 0.29 to 0.74, as shown in Figure 2. The prediction interval ranged from 0.00 to 1.00. Substantial heterogeneity was detected with an I² value of 99 percent and a significant Q test. Individual study estimates varied widely, with prevalence ranging from approximately 32 percent to nearly 87 percent.

Meta-Analysis of Excessive Daytime Sleepiness Prevalence

**Figure 3:**
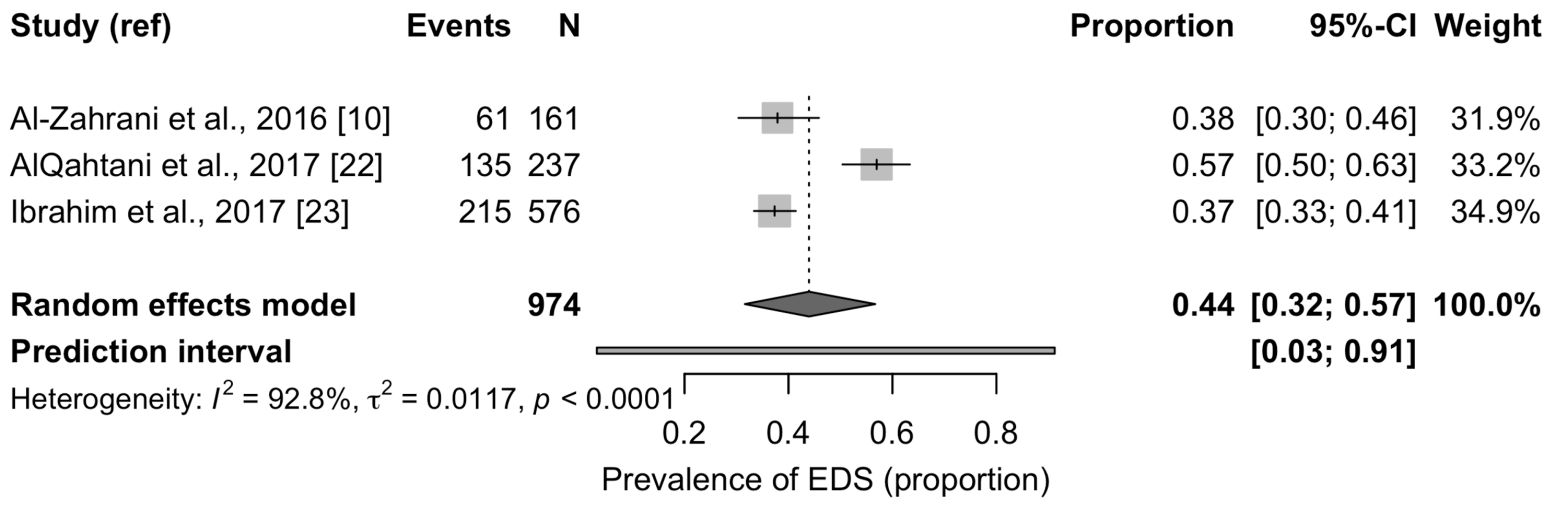
Pooled prevalence of Excessive Daytime Sleepiness (EDS) among medical students in Saudi Arabia

Three studies were included in the meta-analysis of excessive daytime sleepiness, representing a combined sample of 974 students. The pooled prevalence was 0.44 with a 95 percent confidence interval from 0.32 to 0.57, as presented in Figure 3. The prediction interval extended from 0.03 to 0.91. Heterogeneity remained high with an I² value of 92.8 percent and a statistically significant Q statistic. Prevalence estimates across individual studies ranged between 37 percent and 57 percent.

Risk Factors and Academic Performance

Across the included studies, multiple demographic, behavioral, and psychological factors were significantly associated with sleep disturbances among medical students (Table 2). Female gender was a recurring predictor, with the Alrashed study reporting an odds ratio of 1.67 for insomnia [12] and the Ibrahim study noting a higher prevalence of poor sleep among women [23]. Younger students were also more affected, with those aged 21 or younger showing higher odds of poor sleep in the Ibrahim study (OR 2.4) [23]. The academic stage contributed to risk, as preclinical students had an increased likelihood of poor sleep (aOR 1.82) and interns in the Alrashed study demonstrated notably elevated odds of insomnia (OR 3.0) [12]. Psychological distress was one of the strongest contributors, including severe stress (OR 2.81), severe depression (OR 3.10), and morbid anxiety, which showed the highest association in the Ibrahim study (aOR 3.92) [12]. Additional factors included smoking, irregular clinical rotations (OR 3.37), increased screen time, heavy academic workload, and anxiety, with the Mohamed et al. study showing that 91.5 percent of students with severe anxiety experienced insomnia [24].

Findings regarding academic performance demonstrated considerable variability. Several studies reported a negative association between sleep problems and academic outcomes, including the Almansour study [13], where sleep deprivation was significantly linked to lower GPA, and the Rehab Ali Mohamed study [26], where poor sleep quality was associated with poorer academic performance (p < 0.001). In contrast, some studies found no meaningful relationship, such as the Al-Zahrani and Abdelmoaty Goweda studies [10,25], which reported no significant Grade Point Average (GPA) differences based on sleep status. The Ibrahim study produced a contrasting pattern in which students with poor sleep were more frequently represented in higher GPA categories [23].

Quality Assessment of the Included Studies

Quality appraisal using the adapted Newcastle-Ottawa Scale showed that most studies were of moderate methodological quality, with scores between 4 and 6 points (Table 3). Four studies achieved high quality scores of 7 or greater, primarily due to stronger sampling methods and more robust outcome assessments. No studies were classified as low quality. Common limitations included limited control for confounding variables and reliance on self-reported outcomes.

**Table 3:** Quality assessment of the included studies using the Newcastle–Ottawa Scale (NOS) Quality assessment was conducted using the Newcastle–Ottawa Scale (NOS) adapted for observational cross-sectional studies, evaluating three domains: selection (maximum 4 points), comparability (maximum 2 points), and outcome/exposure assessment (maximum 3 points). Studies were classified as high quality (7–9 points), moderate quality (4–6 points), or low quality (0–3 points). Scores reflect methodological rigor based on sampling methods, control of confounders, and validity of Gastroesophageal Reflux Disease (GERD)assessment. The use and structure of the NOS are based on the methodological evaluation described by Stang et al. [18].

Study	Selection (4)	Comparability (2)	Outcome/Exposure (3)	Total (9)	Quality rating
Alshaaer et al., 2012 [11]	2	1	2	5	Moderate
Al-Zahrani et al., 2016 [10]	3	1	2	6	Moderate
AlQahtani et al., 2017 [22]	3	1	2	6	Moderate
Ibrahim et al., 2017 [23]	4	2	3	9	High
Almansour et al., 2020 [13]	3	1	2	6	Moderate
Mohamed et al., 2020 [24]	3	1	2	6	Moderate
Goweda et al., 2020 [25]	3	1	3	7	High
Rehab Ali Mohamed et al., 2020 [26]	3	1	2	6	Moderate
Alrashed et al., 2021 [12]	4	2	3	9	High
Abdulghani et al., 2023 [27]	3	1	2	6	Moderate
Alasimi et al., 2025 [28]	4	1	3	8	High

Discussion

This systematic review and meta-analysis provide an updated synthesis of sleep disorders among medical students in Saudi Arabia and highlight a substantial burden of insomnia, excessive daytime sleepiness, and poor sleep quality in this population. The pooled prevalence estimates for insomnia and excessive daytime sleepiness were 52 percent and 44 percent, respectively, indicating that nearly half of medical students experience clinically significant sleep disruption.

A comparison with international data suggests that the prevalence of sleep disorders among medical students in Saudi Arabia is broadly consistent with, though often higher than, estimates reported in other regions. Studies from the Middle East and North Africa have documented insomnia rates between 30.4 percent in Jordan and 59.1 percent in Morocco, a range similar to that observed in our included studies [29]. These values exceed those reported among medical students in China, where insomnia prevalence has been estimated at approximately 27.8 percent, yet remain lower than figures from Georgia, where insomnia has reached 70 percent in some cohorts [30,31]. Such variation likely reflects differences in academic environments, cultural expectations, living conditions, and stress exposures across countries. Collectively, these comparisons reinforce that sleep disturbances are a global concern among medical trainees, although regional academic pressures and lifestyle factors may amplify their severity in certain settings [5].

Marked variability was observed across the included studies, with reported insomnia prevalence ranging from 32% to nearly 87% [26,28]. This heterogeneity is likely driven not only by differences in study populations and academic environments, but also by the type of sleep assessment instruments used. Studies employing broader screening tools, such as the Pittsburgh Sleep Quality Index and the Sleep-50 Questionnaire, tended to report higher prevalence estimates of sleep problems, often exceeding 70%. These instruments assess multiple dimensions of sleep quality and general sleep disturbances using relatively inclusive cut-off criteria, which may capture a wider spectrum of subclinical and symptomatic sleep problems.

Importantly, the magnitude and direction of variability in prevalence estimates appear to be partly driven by differences in the sleep assessment instruments used across studies. Tools such as the Pittsburgh Sleep Quality Index (PSQI) assess multiple dimensions of sleep quality over a one-month period and apply relatively broad cut-off criteria, which may lead to higher prevalence estimates of poor sleep [6]. In contrast, instruments such as the Insomnia Severity Index (ISI) and Athens Insomnia Scale (AIS) focus more specifically on insomnia symptoms and their perceived severity, potentially yielding lower and more conservative prevalence estimates [6]. Studies using broader screening tools, including PSQI and Sleep-50, tended to report higher prevalence rates, whereas those employing symptom-specific instruments reported comparatively lower rates [23,26]. This methodological variation likely contributes substantially to the observed heterogeneity and suggests that prevalence estimates derived from similar instruments may be more directly comparable and robust.

A consistent pattern across studies was the strong influence of demographic and psychological factors on sleep outcomes. Female students were more likely to report insomnia or poor sleep, which is consistent with global evidence suggesting greater vulnerability to sleep disturbances among women [32]. Younger students and those in early academic years also demonstrated higher sleep disturbance rates, possibly due to adaptation challenges and academic transition stress [33]. Psychological distress emerged as one of the most powerful correlates of sleep difficulties. Severe anxiety, stress, and depressive symptoms were significantly associated with insomnia, with effect sizes in some studies exceeding odds ratios of 3. These findings support the widely documented bidirectional relationship between sleep disruption and mental health disorders and underscore the importance of integrated psychological support within medical education settings [34,35].

The relationship between sleep and academic performance was inconsistent across studies. Some investigations demonstrated clear links between sleep deprivation or poor sleep quality and lower GPA, while others found no meaningful association. One study reported an unexpected pattern in which poor sleepers had higher GPAs [23]. These inconsistencies may reflect differences in academic evaluation systems, self-reporting bias, or compensatory behaviors among high-performing but sleep-deprived students [36]. Nonetheless, the overall evidence suggests that sleep problems have the potential to impair academic functioning and should be considered a key component of academic success strategies.

Taken together, these findings highlight a critical need for targeted interventions to address sleep health among medical students in Saudi Arabia. Educational institutions may consider integrating sleep hygiene programs, stress management workshops, and mental health screening into the curriculum [37,38]. Enhancing awareness of the consequences of poor sleep and promoting early recognition of sleep problems may help reduce their academic and psychological impact. Future research should incorporate longitudinal designs and standardized diagnostic criteria to better define sleep disorder trajectories and clarify causal pathways.

Limitations

This review is limited by the cross-sectional nature of all included studies, which prevents inference about causality between sleep disorders and associated factors. Considerable heterogeneity was present due to variations in assessment tools, study populations, and outcome definitions. All studies relied on self-reported questionnaires, which may introduce recall and reporting bias. The number of eligible studies was small, restricting subgroup analyses and preventing evaluation of publication bias. Additionally, most studies were conducted in single institutions, which may limit the generalizability of findings to all medical students in Saudi Arabia.

## Conclusions

This systematic review and meta-analysis demonstrates a high burden of sleep disorders among medical students in Saudi Arabia, with pooled estimates indicating that roughly half experience insomnia and nearly half report excessive daytime sleepiness. Psychological distress, female gender, younger academic stage, and lifestyle factors were consistently associated with poorer sleep. Although findings regarding academic performance were mixed, inadequate sleep remains a significant concern for student well-being and learning. These results underscore the need for institutional strategies that promote sleep health, integrate mental health support, and encourage early identification of sleep problems to safeguard academic and psychological outcomes.

## References

[1] Sateia MJ (2014). International classification of sleep disorders-third edition: highlights and modifications. Chest.

[2] San L, Arranz B (2024). The night and day challenge of sleep disorders and insomnia: a narrative review. Actas Esp Psiquiatr.

[3] Shah AS, Pant MR, Bommasamudram T (2025). Effects of sleep deprivation on physical and mental health outcomes: an umbrella review. Am J Lifestyle Med.

[4] Canever JB, Zurman G, Vogel F (2024). Worldwide prevalence of sleep problems in community-dwelling older adults: a systematic review and meta-analysis. Sleep Med.

[5] Azad MC, Fraser K, Rumana N, Abdullah AF, Shahana N, Hanly PJ, Turin TC (2015). Sleep disturbances among medical students: a global perspective. J Clin Sleep Med.

[6] Lin Y, Chen Q, Chen Z, Qiu S, Wang L (2025). Evaluation of sleep quality and influencing factors among medical and non-medical students using machine learning techniques in Fujian during the public health emergencies. Front Psychiatry.

[7] Shen Z, Wang T, Zeng X, Wang J (2025). Discrepant associations between psychological stress and sleep quality among medical and non-medical students: a nationwide cross-sectional study in China. Behav Psychol Psicol Conduct.

[8] Benitez A, Gunstad J (2012). Poor sleep quality diminishes cognitive functioning independent of depression and anxiety in healthy young adults. Clin Neuropsychol.

[9] Almalki A, Shehata M, Siddiqui K (2025). Sleep quality among a sample of medical students and the association with academic performance: an updated data. J Epidemiol Glob Health.

[10] Al-Zahrani JM, Aldossari KK, Abdulmajeed I, Al-Ghamdi SH, Al-Shamrani AM, Al-Qahtani NS (2016). Daytime sleepiness and academic performance among Arab medical students. J Thorac Dis.

[11] Eddin Farouq Alshaaer N, Marashli E, Mahgoub M (2012). The prevalence of insomnia in medical students: impact on academic performance. Cureus.

[12] Alrashed FA, Sattar K, Ahmad T, Akram A, Karim SI, Alsubiheen AM (2021). Prevalence of insomnia and related psychological factors with coping strategies among medical students in clinical years during the COVID-19 pandemic. Saudi J Biol Sci.

[13] Almansour A, Aljammaz F, Ahmeda A (2020). The prevalence of sleep deprivation and its influence on students’ life attending medical school at King Saud University. Int J Pharm Phytopharmacol Res.

[14] Cumpston M, Li T, Page MJ, Chandler J, Welch VA, Higgins JP, Thomas J (2019). Updated guidance for trusted systematic reviews: a new edition of the Cochrane Handbook for Systematic Reviews of Interventions. Cochrane Database Syst Rev.

[15] Page MJ, McKenzie JE, Bossuyt PM (2021). The PRISMA 2020 statement: an updated guideline for reporting systematic reviews. BMJ.

[16] Frandsen TF, Bruun Nielsen MF, Lindhardt CL, Eriksen MB (2020). Using the full PICO model as a search tool for systematic reviews resulted in lower recall for some PICO elements. J Clin Epidemiol.

[17] Ouzzani M, Hammady H, Fedorowicz Z, Elmagarmid A (2016). Rayyan-a web and mobile app for systematic reviews. Syst Rev.

[18] Stang A (2010). Critical evaluation of the Newcastle-Ottawa scale for the assessment of the quality of nonrandomized studies in meta-analyses. Eur J Epidemiol.

[19] Abdulmajeed J, Chivese T, Doi SA (2025). Overcoming challenges in prevalence meta-analysis: the case for the Freeman-Tukey transform. BMC Med Res Methodol.

[20] Lau J, Ioannidis JP, Terrin N, Schmid CH, Olkin I (2006). The case of the misleading funnel plot. BMJ.

[21] Sterne JA, Sutton AJ, Ioannidis JP (2011). Recommendations for examining and interpreting funnel plot asymmetry in meta-analyses of randomised controlled trials. BMJ.

[22] AlQahtani MS, Alkhaldi TM, Al-Sultan AM, Bin Shihah AS, Aleid AS, Alzahrani ZK, Alfaryan KH (2017). Sleeping disorders among medical students in Saudi Arabia in relation to anti-insomnia medications. Egypt J Hosp Med.

[23] Ibrahim NK, Badawi FA, Mansouri YM, Ainousa AM, Jambi SK, Fatani AN (2017). Sleep quality among medical students at King Abdulaziz University: a cross-sectional study. J Community Med Health Educ.

[24] Mohamed EY, Abdulrahim SA, Sami W (2020). Insomnia and related anxiety among medical students. J Res Med Dent Sci.

[25] Abdelmoaty Goweda R, Hassan-Hussein A, Ali Alqahtani M (2020). Prevalence of sleep disorders among medical students of Umm Al-Qura University, Makkah, Kingdom of Saudi Arabia. J Public Health Res.

[26] Mohamed R, Alotaibi M, Almutairi N (2020). The prevalence of sleep disturbances among medical college students of Jouf University and its effect on academic achievement. Int J Med Dev Ctries.

[27] Abdulghani HM, Marwa K, Alghamdi NA (2023). Prevalence of the medical student syndrome among health professions students and its effects on their academic performance. Medicine (Baltimore).

[28] Alasimi AH, Brandenberger KJ, Zimmerman RD, Shan SH (2025). Insomnia symptoms and their association with academic performance among respiratory therapy students in Saudi Arabia and the United States. Sci Rep.

[29] Essangri H, Sabir M, Benkabbou A (2021). Predictive factors for impaired mental health among medical students during the early stage of the COVID-19 pandemic in Morocco. Am J Trop Med Hyg.

[30] Solanki S, Venkiteswaran A, Saravanabawan P (2023). Prevalence of insomnia and factors influencing its incidence in students of Tbilisi State Medical University: a cross-sectional study. Cureus.

[31] Zhang M, Qin L, Zhang D (2023). Prevalence and factors associated with insomnia among medical students in China during the COVID-19 pandemic: characterization and associated factors. BMC Psychiatry.

[32] Schmickler JM, Blaschke S, Mess F, Olson N, Reiner B, Schulz T, Friedrich J (2025). Sleep in the academic sphere: identifying sleep profiles and their influencing factors using latent profile analysis in German university students. BMC Psychol.

[33] Minghelli B (2022). Sleep disorders in higher education students: modifiable and non-modifiable risk factors. North Clin Istanb.

[34] Hyun S, Hahm HC, Wong GT, Zhang E, Liu CH (2021). Psychological correlates of poor sleep quality among U.S. young adults during the COVID-19 pandemic. Sleep Med.

[35] Kaczkurkin AN, Tyler J, Turk-Karan E, Belli G, Asnaani A (2021). The association between insomnia and anxiety symptoms in a naturalistic anxiety treatment setting. Behav Sleep Med.

[36] Baklola M, Terra M, Al-barqi M (2024). Prevalence of insomnia among university students in Saudi Arabia: a systematic review and meta‑analysis. Egypt J Neurol Psychiatry Neurosurg.

[37] A Mariappan V, Mukhtar F (2025). Psychological interventions for sleep problems among medical and paramedical students: a systematic review. J Clin Psychol.

[38] Duthie CJ, Cameron C, Smith-Han K (2023). Sleep management strategies among medical students at the University of Otago. Behav Sleep Med.

